# A Comparative Study of Physicochemical, Aroma, and Color Profiles Affecting the Sensory Properties of Grape Juice from Four Chinese *Vitis vinifera* × *Vitis labrusca* and *Vitis vinifera* Grapes

**DOI:** 10.3390/foods13233889

**Published:** 2024-12-02

**Authors:** Chen Yang, Xinyue Fan, Fei Lao, Jin Huang, M. Monica Giusti, Jihong Wu, Hongmei Lu

**Affiliations:** 1 College of Food Science and Nutritional Engineering, China Agricultural University, National Engineering Research Center for Fruit & Vegetable Processing, Key Laboratory of Fruit and Vegetable Processing, Ministry of Agriculture and Rural Affairs, Beijing Key Laboratory for Food Non-Thermal Processing, Beijing 100083, China; ychen5716@163.com (C.Y.); huangjin202110@163.com (J.H.); wjh7268@cau.edu.cn (J.W.); 2 Department of Food Science and Technology, The Ohio State University, Columbus, OH 43210, USA; giusti.6@osu.edu; 3 Institution of Fruit Technology Guidance of Guangxi Zhuang Autonomous Region, Nanning 530022, China; lhm2902@163.com

**Keywords:** grape juice, variety, aroma, color

## Abstract

In order to compare the grape juice sensory properties of four common seedless grape varieties (Kyoho, Summer Black, Moldovan, and Sweet Sapphire) in China, a thorough comparison of these grape varieties was conducted. Physicochemical indicators, aroma, and color characteristics were analyzed and compared to a commercially available not-from-concentrate Concord grape juice. The contents of fructose, glucose, and seven organic acids were evaluated. Kyoho and Sweet Sapphire possessed optimal Brix–acid ratio in the range around 35–40. In terms of aroma, 60 volatiles were identified by GC-MS, including 16 alcohols, 9 terpenes, 6 aldehydes, and 4 ketones. Kyoho demonstrated the highest aroma intensity with superior floral and fruity notes, while Sweet Sapphire showed the lowest aroma intensity with a grassy scent. Additionally, grape pigment profiles were investigated by HPLC-PDA-MS. Summer Black grapes featured a vibrant color, and 52.5% of their anthocyanins were acylated, which helps provide good stability in follow-up processing. Concord juice showed the best overall properties, with the most saturated color and delightful aroma. It was suggested that blending Summer Black with Kyoho could be a promising way to achieve balanced color, taste, and aroma of grape juice. This study provides a feasible and promising combination of Chinese local grapes for making all-round high-quality juice products.

## 1. Introduction

In recent years, China’s grape production has experienced continuous growth, accompanied by notable enhancements in quality and variety optimization [1]. China’s total grape production attained 12.6 million tons in 2022, elevating China to be the world’s foremost grape producer [2]. However, unlike other prominent grape-producing countries where 73% of grapes are allocated for wine, grape juice, and other processed goods, with only 27% consumed fresh [1], over 80% of Chinese grapes are consumed fresh, while less than 10% are designated for juice production [3].

Grape juice remains one of the most preferred grape products, highly esteemed by consumers due to its appealing color, distinctive aroma, and pleasant flavor [4]. Besides providing essential nutrients such as sugars, vitamins, and minerals, grape juice is rich in polyphenols with antioxidant properties, including anthocyanins, flavanols, procyanidins, and phenolic acids [5,6,7]. These polyphenols offer numerous potential health benefits, reducing the risk of inflammatory responses and their related diseases [8,9,10].

China imported 37,846 tons of grape juice (including both concentrated and non-concentrated juices) in 2021, representing 12% of the total imported juice (Chinese Customs, 2021). Although China has a vast consumer market for grape juice, the advancement of the grape juice processing industry is still relatively slow, lacking significant production capacity. Concord grape, an America-originated cultivar derived from *Vitis labrusca*, has been widely used in making grape juice [11]. However, Concord grape is not the main cultivar used in China. Instead, most domestically produced grape juice utilized varieties suitable for both fresh consumption and juicing, such as Kyoho and Summer Black, which are hybrids between *Vitis vinifera* and *Vitis labrusca* [3]. These products lack distinctive flavor characteristics and core competitiveness, ultimately failing to satisfy consumer demand. Evaluation of grape varieties and their juice-making properties to achieve a suitable combination of Chinese varieties for grape juice is of great interest for local industry.

Currently, research on grape juicing adaptability primarily focuses on specialized juicing varieties, such as Concord. Information on varieties that are good for both fresh consumption and juicing is relatively limited. This study selected four popular seedless purple table grapes commonly found in the Chinese market, including Kyoho and Summer Black, which have been used for juicing in China, to identify suitable combinations of varieties for grape juice production in China. The differences in fundamental physicochemical indicators, color components, and aroma profiles of grape juice among different varieties were investigated to gain a better understanding of local grapes. This study provides insights into their compositional differences that are related to their sensory properties and offers valuable references for the production of high-quality grape juice featuring specific sensory superiority.

## 2. Materials and Methods

### 2.1. Materials

Summer Black, Moldovan, Kyoho, and Sweet Sapphire were purchased from local farms (Table 1). A commercial bottled 100% Concord (*Vitis labrusca*) grape juice was obtained from Whole Foods Market (Austin, TX, USA) in January 2024. All grape samples were maintained at −20 °C prior to analysis.

### 2.2. Chemicals and Reagents

HCl was purchased from Sinopharm Chemical Reagent Co., Ltd. (Beijing, China). Phosphoric acid, methanol, acetonitrile, and acetic acid of high-performance liquid chromatography (HPLC) grade were purchased from Thermo Fisher (Shanghai, China). KCl, sodium metabisulfite, sodium acetate, sodium carbonate, metaphosphoric acid, and formic acid of mass spectrometry (MS) grade and MS-grade acetonitrile were purchased from Macklin (Shanghai, China). Tartaric acid, malic acid, citric acid, oxalic acid, succinic acid, and L-ascorbic acid were purchased from Yuanye Bio-Technology Co., Ltd. (Shanghai, China). Sucrose, fructose, glucose, Folin and Ciocalteu’s phenol reagent (1 N), potassium dihydrogen phosphate, and gallic acid were from Solarbio (Beijing, China). Sodium hydroxide standard solution (0.1 M) was purchased from Beijing Institute of Chemical Reagents Co., Ltd. (Beijing, China). 2-Octanol was purchased from TCI Chemical Industry (Tokyo, Japan). Unless specified, all reagents were of analytical grade.

### 2.3. Grape Juice Preparation

Uniform grapes (200 g) were selected, cleaned, de-stemmed, and blended in a mixer (model MJ-BL25B3, Guangdong Midea Life Electric Appliance Manufacturing Co., Ltd., Foshan, China). The juice was subsequently filtered through four layers of gauze and collected. The residue was squeezed for remaining juice, which was then also collected. The total mass of the extracted juice was accurately measured. These steps were repeated three times for each variety.

### 2.4. Juice-Making Quality Evaluation

#### 2.4.1. Physiochemical Properties

Yield was calculated by dividing the total mass of the filtered grape juice by the mass of the grapes used. pH value was determined using a pH meter (FiveEasy Plus, Mettler Toledo Technology Co., Shanghai, China). The soluble solids content was measured using an Abbey refractometer (DR-A1, Atago Scientific Instrument Co., Ltd., Guangzhou, China). Titratable acidity was assayed by titrating to pH 8.2 using a potentiometric titrator (842 Titrando, Aptar China Co., Ltd., Shanghai, China) with 0.1 M NaOH. The Brix–acid ratio was determined by dividing the soluble solids content (°Brix) by the titratable acid content (g/L).

Sugars (glucose, fructose, sucrose): The contents of glucose, fructose, and sucrose in grape juice were determined using the HPLC method described by Coelho et al. with modification [12]. Grape juice was diluted with a 10% aqueous acetonitrile solution and aspirated through an m-PFC multiplug filtration clean-up (Beijing KNORTH Technology Co., Ltd., Beijing, China) connected with a 0.22 μm polyethersulfone microporous filter membrane. The purified solution was collected and analyzed using an HPLC (SPD-20A, Shimadzu, Shanghai, China) with an evaporative light scattering detector. A Decoma Polyamino HILIC column (250 mm × 4.6 mm, 5 μm, Dikema Technology Co., Ltd., Beijing, China) equipped with a guard column was at 40 °C. The injection volume was 10 μL, and the flow rate was 1.0 mL/min. Mobile phases were ultrapure water (A) and acetonitrile (B), with a gradient elution program: 0–8 min, 25% B; 8–10 min, 25–30% B; 10–20 min, 30% B; 20–22 min, 30–25% B; 22–25 min, 25% B. Quantification of sugars was performed by 5-point standard curves of glucose, sucrose, and fructose ranging from 0.02 to 2 g/L. The sweetness index was computed based on the following equation [13]:

Sweetness index = (glucose × 0.75) + (fructose × 1.75) + sucrose(1)

Organic acids (oxalic acid, tartaric acid, malic acid, acetic acid, citric acid, and succinic acid): The HPLC methods described in Chinese national food safety standard GB 12456 [14] and Güler et al. [15] were adapted with modification. Specifically, grape juice was diluted with ultrapure water in a centrifuge tube, followed by centrifugation at 10,000 rpm for 10 min at 4 °C. The supernatant was filtered through a 0.22 μm polyethersulfone membrane and transferred to a sample vial. Analysis was performed using an HPLC (SPD-20A, Shimadzu) with a UV detector and Athena C18 column (120 Å, 250 mm × 4.6 mm × 5 µm, ANPEL Laboratory Technologies Inc., Shanghai, China), utilizing a mobile phase of 0.025 M potassium dihydrogen phosphate adjusted to pH 2.6 with phosphoric acid, at a flow rate of 1.0 mL/min, an injection volume of 20 μL, and a column temperature maintained at 40 °C. The detection wavelength was set at 210 nm. Quantification was based on standard curves derived from the standard substances of oxalic acid, tartaric acid, malic acid, acetic acid, citric acid, and succinic acid ranging from 0.01 to 3 g/L.

Ascorbic acid: Ascorbic acid was determined by the HPLC method of Chinese national food safety standard GB 5009.86 [16] with slight modifications. Specifically, grape juice was diluted with metaphosphoric acid solution (20 g/L) in a centrifuge tube, followed by centrifugation at 10,000 rpm for 10 min at 4 °C. The supernatant was filtered through a 0.22 μm polyethersulfone membrane and transferred to a sample vial. Analysis was performed using an HPLC (SPD-20A, Shimadzu) with a UV detector and Athena C18 column (120 Å, 250 mm × 4.6 mm × 5 µm, Shanghai ANPEL Laboratory Technologies Inc.), utilizing a mobile phase of 0.05 M potassium dihydrogen phosphate adjusted to pH 2.4 with phosphoric acid, at a flow rate of 1.0 mL/min, an injection volume of 20 μL, and a column temperature maintained at 40 °C. The detection wavelength was set at 245 nm. Quantification was based on standard curves derived from the standard substance of ascorbic acid ranging from 0 to 100 mg/L.

#### 2.4.2. Volatile Composition

Aroma compounds were enriched using solid-phase microextraction combined with GC-MS as described by Zhang et al. [17]. Briefly, 5 mL of grape juice was added to a 20 mL headspace vial (ANPEL Laboratory Technologies Inc., Shanghai, China) containing 2 g of sodium chloride, vortex mixed, and then 10 μL of 2-octanol (8.22 μg/L) was added as an internal standard. The headspace vial was incubated at 40 °C with shaking for 20 min before being placed at the inlet of the GC-MS system for desorption. GC-MS analysis was performed using an Agilent 7890 gas chromatograph equipped with an Agilent 5975C series mass spectrometer (Agilent Technologies, Santa Clara, CA, USA) and a DB-Wax capillary column (Agilent Technologies, Santa Clara, CA, USA). The oven temperature was held at 45 °C for 5 min, ramped at 5 °C min^−1^ to 100 °C and held for 5 min, then ramped to 200 °C at 10 °C min^−1^ and held for 5 min, and finally ramped to 250 °C at 10 °C min^−1^ for 5 min. MS settings were similar to those described in the literature, with a mass scan range of 30–500 *m*/*z*. Volatiles were identified by matching mass spectra with the NIST 17 database. The contribution of volatile aroma compounds to the overall flavor was determined by calculating the odor activity value (OAV). The compounds with OAV ≥ 1 were considered to have aroma activity. The aroma threshold value referred to the literature [18].

#### 2.4.3. Pigment Composition and Color Properties

Monomeric anthocyanin: Monomeric anthocyanin content was determined by the pH differential method [19]. Briefly, grape juice was diluted with pH 1.0 and 4.5 buffers. After equilibrating for 30 min in darkness, the absorbances at 700 nm and 520 nm were determined by a UV–visible spectrophotometer (UV 1800 model, Shimadzu Laboratory Equipment Co.). Monomeric anthocyanin content was calculated as cyanidin-3-glucoside equivalents with a molecular weight of 449 g/mol and a molar absorptivity of 26,900 L/(mol × cm).

Anthocyanin profile: Anthocyanins were purified by solid-phase extraction (VisiprepTM DL, Supelco, Bellefonte, PA, USA) [20,21]. The C18 cartridge (Waters Corporation, Milford, MA, USA) was activated by methanol and washed with acidified water (0.01% HCl) Then, grape juice was loaded on the cartridge and washed with 0.01% acidified water. Anthocyanins were then eluted with acidified methanol (0.01% HCl) and methanol was removed by a nitrogen evaporator (PGN-16L, Beijing Pulaixi Technology Co., Ltd., Beijing, China). After that, the sample was dissolved by 0.01% HCl water and then filtered through a 0.22 μm polyethersulfone membrane for further analysis.

Anthocyanins were identified by the UPLC-PDA-MS/MS method with minor modification from Wang et al. [22] and Wu et al. [23]. The UPLC (ACQUITY UPLC I-Class/Xevo TQ-S, Waters, USA) was equipped with PDA and MS/MS detectors. Separation was performed on an ACQUITY UPLC BEH C18 130Å column (2.1 mm × 100 mm × 1.7 µm, Waters, USA) at 40 °C. The injection volume was 5 μL, the flow rate was 0.25 mL·min^−1^, and the detection wavelength was 520 nm. Mobile phases consisted of 4.5% formic acid in water (A) and acetonitrile (B), with a gradient elution program set as follows: 0–2 min, 5% B; 2–30 min, 5–20% B; 30–35 min, 20–30% B; 35–38 min, 30–50% B; 38–41 min, 50% B; 41–43 min, 50–5% B; 43–48 min, 5% B. The Xevo TQ-S was operated in ESI positive ion mode with specific parameters. The MS scan range was 200–2000 *m*/*z*. Parent scan selected six anthocyanidins with specific *m*/*z* values of 271, 287, 301, 303, 317, and 331, and natural loss scan selected fragments representing potential groups in grape anthocyanin fragments of 132, 146, 162, 176, 206, 308, 324, 470, and 616.

Phenolics: Total phenolic content was determined by the Folin–Ciocalteu colorimetric method [24]. Briefly, 3 mL 0.1 N Folin and Ciocalteu’s phenol reagent was added to 20 μL sample and mixed thoroughly, followed by 5 mL 10% sodium carbonate solution. After incubating in the dark at room temperature for 60 min, the absorbance was measured at 765 nm using a UV-Vis spectrophotometer (UV 1800, Shimadzu). Phenolics content was calculated as gallic acid equivalents and quantification was based on standard curves derived from the gallic acid standard diluted at concentrations ranging from 0 to 5 mg/L.

Polymeric anthocyanin: Polymeric anthocyanin was determined by the sodium bisulfite bleaching method [19]. Briefly, 1.4 mL sample was mixed with 0.1 mL of sodium bisulfite solution (prepared by dissolving 1 g sodium metabisulfite in 5 mL distilled water) or distilled water and incubated in the dark for 15 min. Absorbance was measured at 420 nm, 520 nm, and 700 nm using a microplate reader (Spark 10 M, Tecan, Männedorf, Switzerland). The color density (treated with distilled water), polymeric color (treated with sodium bisulfite solution), and percent polymeric color were computed based on the following equations [19]:

Color density = [(A_420nm_ − A_700nm_) + (A_520nm_ − A_700nm_)] × DF (2)

Polymeric color = [(A_420nm_ − A_700nm_) + (A_520nm_ − A_700nm_)] × DF (3)

Percent polymeric color (%) = (polymeric color/color density) × 100(4)

Color: The juices were centrifuged at 10,000 rpm, 4 °C for 10 min and the supernatants were collected for measurement. The supernatant was placed in a glass cuvette with a a 5 mm optical path length and scanned using a UV–visible spectrophotometer (UV 1800, Shimadzu) within the wavelength range of 380 nm to 780 nm. The obtained spectra data were imported into the ColorBySpectra software (2017 version) [25] to derive the color data. Colorimetric values were expressed by CIELAB (L*, a*, b*, and L*, C*_ab_, h_ab_) values. The color data were input into the Adobe Color website [26] to generate the corresponding color swatches for better visual comparison.

### 2.5. Statistical Analysis

All experiments were conducted in triplicates, with results presented as the mean ± standard deviation of the three replicates. The significance of differences among samples was determined using one-way ANOVA with Origin 2024 (*p*-value < 0.05). Graphical representations were created using Origin 2024 software.

## 3. Results and Discussion

### 3.1. Physicochemical Properties Analysis

#### 3.1.1. Yield, pH, Titratable Acidity, and Soluble Solids

Table 2 presents the physicochemical indicators of the five grape varieties examined in this study, which exhibits significant variations. The juice yield of the four grape varieties analyzed ranged from 69.88% to 76.06%. Notably, Summer Black produced a considerably lower yield in comparison to Kyoho and Sweet Sapphire, potentially attributed to its thicker skin.

Titratable acidity of the grape varieties ranged from 3.20 to 5.15 g/L (calculated as tartaric acid equivalents), with pH values ranging from 3.59 to 3.99 (Table 2). Summer Black exhibited the lowest titratable acidity at 3.20 g/L and the highest pH of 3.99, whereas Concord showed the second-highest titratable acidity at 5.15 g/L and the lowest pH of 3.59. All grape varieties analyzed exhibited soluble solids surpassing 14°Brix, complying with the Chinese national standard GB/T 31121 [27] that mandates a soluble solids content exceeding 11°Brix for grape juice. Notably, Summer Black and Moldovan had the highest soluble solids contents compared to the other varieties, measuring 22.8 and 21.8°Brix, respectively.

The Brix–acid ratio exhibits a strong correlation with consumer acceptance and provides an effective means of assessing consumer preferences for fruits, vegetables, and their associated products [28]. The Brix–acid ratio emerges as a more appropriate predictor of consumer preference specifically for seedless table grapes than soluble solids or titratable acidity considered individually, and a previous study suggested that an optimal Brix–acid ratio for seedless table grapes ranges between 35 and 40 [29]. The Brix–acid ratio of the five grape juices examined in this study ranged from 27.84 to 71.66, wherein Summer Black exhibited the highest value (71.66) and Kyoho the lowest (27.84). Sweet Sapphire, with a ratio of 41.74, was the closest to the optimal sugar–acid ratio range, followed closely by Concord (31.56). It is noteworthy that Kyoho and Concord demonstrated no significant differences regarding pH, titratable acidity, and Brix–acid ratio.

#### 3.1.2. Sugar, Organic Acid, and Sweetness

The composition and content of soluble sugars and organic acids have a significant impact on grape quality, as their ratio may affect the sweet–sour balance of the juice drinking experience. Among the five grape varieties, the highest fructose content was observed in Sweet Sapphire (91.14 g/L), while the lowest was in Concord (60.88 g/L). Similarly, the highest glucose content was found in Summer Black (64.89 g/L), and the lowest in Concord (36.58 g/L). Sucrose was not detected in any of the five varieties (Table 2). Consistent with a previous study, the soluble sugar components in grapes are predominantly glucose and fructose, while sucrose exists only at trace levels [28].

The sweetness intensity of fruit and vegetable juices is not only related to the total sugar content but also to the sugar profile. By calculating the sweetness index using the equation previously provided (Equation (1)), it was found that the highest sweetness was observed in Moldovan (205.14), while the lowest was in Concord (133.98). There were no significant differences in glucose, fructose, and sweetness among Summer Black, Moldovan, and Sweet Sapphire, nor were significant differences found in these three indicators between Kyoho and Concord (Table 2).

Significant variations were observed in the composition of organic acids across the five varieties (Table 2). Tartaric acid was the most prevalent organic acid in grapes (Table 2), ranging from 49.5% to 87.0% of the total organic acid, with Summer Black having the highest content (19.26 g/L) and Concord the lowest (4.6 g/L). Another prominent organic acid was malic acid, constituting 5.5% to 30.8% of the total organic acids, with Concord having the highest (2.80 g/L) and Moldovan the lowest (1.05 g/L). Significant variations existed in the levels of ascorbic acid, acetic acid, citric acid, succinic acid, and oxalic acid (Table 2). Similar to previous grape studies, the primary organic acids in grapes were tartaric acid and malic acid, which collectively account for over 90% of the total organic acid content [28,30]. Organic acids are major metabolites in grapes that not only participate in various physiological and biochemical reactions but also play a crucial role in shaping the unique flavor characteristics of grapes by influencing their pH, acidity, and chemical stability [28,31].

#### 3.1.3. Summary of Physicochemical Properties

The juice yield of 76.06 ± 2.52% from Sweet Sapphire was the highest of all, though no significant difference was observed in the juice yields among Moldovan, Kyoho, and Sweet Sapphire. Summer Black yielded the least juice (69.88 ± 1.39%). The soluble sugar contents exhibited relatively minor variations among different grape varieties. The organic acid components demonstrated substantial differences among various grape varieties. Kyoho and Sweet Sapphire exhibited the Brix-acid ratio closest to the optimal range of 35 to 40, in contrast to Summer Black and Moldovan, which possessed a Brix–acid ratio exceeding 60, leading to a sweeter tasting profile.

### 3.2. Aroma Compounds Analysis

#### 3.2.1. Classes and Concentrations of Aroma Compounds

Significant variations were observed in the total aroma content and composition among the five grape varieties (Table 3). Kyoho (1972.54 μg/L) and Concord (1774.61 μg/L) exhibited notably higher aroma compound concentrations compared to other varieties, while Sweet Sapphire (409.02 μg/L) had the lowest (Table 3). Among five grape varieties, 60 volatile flavor components were identified, including 16 alcohols, 9 terpenes, 6 aldehydes, 4 ketones, 1 acid, 12 esters, 9 aromatics, 1 norisoprenoid, and 2 other flavor components (Table 4). Similar to a previous study on aroma profiles of 20 Chinese table grape cultivars, alcohols, aldehydes, and esters were the dominant volatiles in quantity among different table grape pulps [32]. Terpenes were another major volatile class, but the majority were more concentrated in grape skin rather than the pulp [32].

The contents of alcohols were relatively high in all grapes (Table 3). Alcohols were the primary class of aroma in Summer Black, Moldovan, and Sweet Sapphire, which accounted for 36.28–50.48% of the total volatiles. The total alcohols in table grapes can be as high as 57,763.94 μg/L (cv Hutai No. 8) [33] and as low as 244.32 μg/kg (cv Suiho) [32]. The grape juice alcohol contents in this study appeared to be significantly lower than the levels reported by Yao et al. [33] and the majority of Chinese table grapes [32] but were comparable to those reported in several Chinese table grape cultivars [32] and Muscadine grape (Vitis rotundifolia Michx cv. Carlos) juice [34]. Besides the variation due to varieties, the discrepancy might be attributed to differences in the juice extraction method. In this study, as well as the Muscadine grape juice study [34], grapes were directly juiced and filtered at room temperature (25 °C), whereas the alcohol-rich studies juiced and centrifuged at a refrigerated temperature [32,33]. The Hutai No. 8 grape study also added D-gluconolactone and polyvinyl pyrrolidone to stabilize the juice matrix [33]. This methodological difference could have played a role in the variation among the levels of aroma compounds. Additionally, it should be noted that the grape pomace still retained grape-like aroma after filtration, suggesting a portion of the aroma might have remained in the pomace and failed to transfer to the juice. This matched a previous apple pomace hydrodistillate study, which found intense peaks of volatile alcohols in the apple pomace [35].

Esters were the primary aroma compounds in Kyoho and Concord (accounting for 85.12% and 82.38%, respectively), while they only accounted for 1.44% in Sweet Sapphire. The concentrations of esters among the five grape varieties were 5.88–1554.26 μg/L, indicating significant cultivar variation. The variation of ester content among different grapes has been widely reported. The ester content in fresh Turkish black grape (*Vitis vinifera*) juice was 49.48 ± 3.32 μg/kg [36], the total esters in 20 Chinese table grapes ranged from 0.37 μg/kg (cv High Bailey) to 4878.55 μg/kg (cv Jumeigui) [32]. Among which, ethyl acetate was identified in all five varieties and accounted for 92.40–100% of the esters, being the most prominent ester in grapes (Table 4). Ethyl acetate was also reported as the dominant ester in Kyoho [32] and Hutai No. 8 [33], constituting over 90% of the total ester content. Ethyl acetate features typical fruity aroma and is widely found in fruit juices such as orange [37], peach [38], mango [39], and pineapple [40].

Norisoprenoid was only found in Kyoho and Concord, with a concentration slightly above 3 μg/L (Table 3). β-Damascenone was the norisoprenoid identified in Kyoho and Concord (Table 4), which provides a strong rose and apple-like aroma [32,33]. The contents of β-damascenone in other Chinese table grapes were around 0.01–0.18 μg/kg [32], much lower than that of Kyoho and Concord. Since the content of norisoprenoid varies a lot among varieties, it could be a great indicator to classify grapes into different aroma categories.

#### 3.2.2. Odor Activity Values

**Figure 1:**
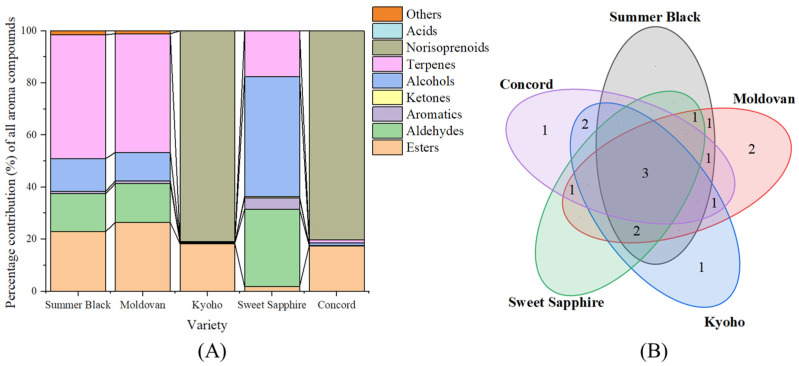
Percentage contributions of each class of aroma components (**A**) and Venn diagram of aroma-active components (**B**). The numbers in (**B**) represented the number of compounds in each respective region, with overlaps indicated intersection between the sets.

A total of 16 aroma-active compounds (OAV ≥ 1) were identified across the five grape varieties (Table 4), primarily consisting of esters, terpenes, alcohols, and norisoprenoids. The aroma-contributing compounds varied significantly among the different grape varieties (Figure 1A). Terpenes were the primary aroma contributors in Summer Black and Moldovan (47.54% and 45.54%, respectively), norisoprenoids dominated in Kyoho and Concord (80.92% and 80.23%, respectively), while alcohols (45.97%) and aldehydes (29.63%) were the main contributors in Sweet Sapphire (Figure 1A). Ethyl acetate (fruity, pineapple-like note) [41], 1-hexanol (green, grass note) [42], and linalool (sweet, grape-like note) [43] were identified as the three common aroma-active compounds across the five grape varieties (Figure 1B, Table 4). Ethyl hexanoate (fruity, green–apple note) [44] was the unique aroma-active compound in Kyoho (Figure 1B, Table 4). Methyl anthranilate was the unique aroma-active compound in Concord (Figure 1B, Table 4), which exhibited typical grape and foxy aromas, serving as the source of the unique foxy and grape-like aroma in American grapes [45,46]. Isovaleraldehyde (apple-like odor) [47] and 1-nonanal (fat, citrus, green note) [48] were the unique aroma-active compounds in Moldovan (Figure 1B, Table 4).

The strong rose, honey, sweet, and cooked apple-like norisoprenoid compound [32,33] β-damascenone contributed over 80% of the overall aroma perception in Kyoho and Concord (Figure 1A). It was present at low concentrations but contributed significantly to the aroma due to its low odor threshold (OAV > 1500). β-Damascenone was also identified as a critical aromatic compound in *Vitis labrusca* and certain hybrid varieties [3,33]. β-Damascenone was suggested to be an important aroma component in American blood grape varieties such as Concord [33], yet the presented study found that the similar *V. vinifera* × *V. labrusca* cultivars, Summer Black and Moldovan, did not show similar levels of β-damascenone to that of Kyoho, suggesting β-damascenone was not unique to American blood grape varieties. Esters were another major aroma contributor in terms of Kyoho and Concord, and ester compounds’ OAVs (OAVs > 300) were notably higher than those in other varieties (Table 4). Besides ethyl acetate, the OAVs of ethyl butyrate and ethyl hexanoate in Kyoho and Concord were much higher than those of the other grape varieties. Both ethyl butyrate and ethyl hexanoate were identified as key aroma compounds in Black Beet grape, Kyoho grape, Jingya grape, and Jumeigui grapes [32]. Esters usually provide rich floral and tropical fruity notes to grapes, and they were the second biggest aroma contributors for Summer Black and Moldovan (Figure 1A).

Represented by linalool and geraniol, terpenes were the most significant compounds in Summer Black and Moldovan (OAVs > 50), contributing floral, fruity, and citrus-like aromas [49]. Terpenes are believed to be one of the main compounds contributing to the typical aroma of Muscat grapes [36]. But the aromatic contribution of terpenes in Kyoho in the present study was the lowest (OAV = 3.49) among the five grapes (Table 4). Similar results were also found in Kyoho pulp juice (OAV< 0.01) and Kyoho skin (OAV = 0.05) [32]. Alcohols and aldehydes were other aroma-contributing compounds for Summer Black and Moldovan (Figure 1A), with hexanal and 1-hexanol being the top-contributing alcohol and aldehyde (Table 4). A rose oxide compound, (2S,4R)-cis-4-methyl-2-(2-methyl-1-propenyl) tetrahydropyran, was identified as an aroma-active substance in Summer Black (OAV = 2.11) and Moldovan (OAV = 1.37) and imparted a unique rose aroma. This rose oxide compound was previously reported in litchi wine to give elegant floral, lychee-like, and rose flavors [50,51].

Sweet Sapphire exhibited the fewest aroma compound types and lowest aroma contribution among all grape varieties (Table 4). The primary aroma contributors in Sweet Sapphire were alcohols, mainly derived from 1-hexanol and isoamyl alcohol, exhibiting grassy and mild sweet aromas [42], followed by aldehydes (hexanal, OAV = 19.2) and terpenes (linalool and geraniol, OAV = 8.16 and 3.45) (Figure 1A, Table 4). The floral and citrus-like linalool and geraniol accumulated as the grapes matured [33]. The OAV of linalool in table grapes can be as high as 54.69 in Shine Muscat grape and was not detectable in Zuijinxiang, Heibaladuo, and Black Swan grapes [32], suggesting notable variation among different cultivars. Ketones and acids exhibited low aroma intensity (OAVs < 1) in all varieties due to their high odor thresholds.

#### 3.2.3. Principal Component Analysis Based on Physicochemical and Aroma Properties

**Figure 2:**
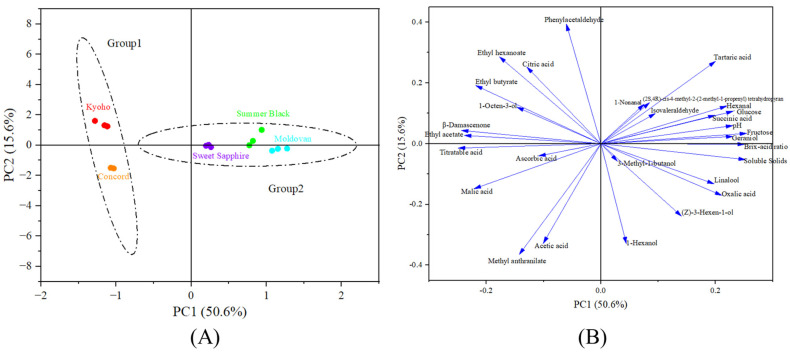
Score plot (**A**) and loading plot (**B**) of five grape varieties based on physicochemical and aroma properties.

This study utilized 13 physicochemical indicators and 16 aroma-active compounds (OAV ≥ 1) of grapes as variables and used principal component analysis (PCA) to classify the five grape varieties into two groups based on these indicators (Figure 2A). The first group consisted of Kyoho and Concord, and the second group consisted of Summer Black, Moldovan, and Sweet Sapphire. PCA revealed clear distinctions among the five grape varieties, with the variance contribution rates of the first six principal components being 50.6%, 15.6%, 14.2%, 11.4%, 3.4%, and 1.9%, respectively, resulting in a cumulative variance contribution of 97.0%. The first two principal components (PC1 and PC2) collectively explained 66.2% of the variance contribution rate.

Figure 2B presents the loading values of indicators on PC1 and PC2. PC1 accounted for the largest variation in the data, showing positive correlations with fructose (loading of 0.255), soluble solids (0.252), Brix–acid ratio (0.251), and glucose (0.231) and a negative correlation with titratable acidity (−0.249). This suggested that PC1 may primarily relate to taste and flavor properties such as sweetness and sourness. PC2 explained the second largest variation in the data, exhibiting positive correlations with phenylacetaldehyde (0.394) and a negative correlation with 1-hexanol (−0.434) and methyl anthranilate (−0.366). This indicated that PC2 may primarily relate to the aroma quality of grape juice. Tartaric acid content exhibited significant loadings (absolute loading values > 0.2) on both PC1 and PC2, indicating it had important influence on the overall quality characteristics of grape juice and may be related to both taste and aroma. All sweetness-related indicators (fructose, glucose, soluble solids content) made significant positive contribution to PC1 (loading > 0.2) and had minimal contribution to PC2. Acidity-related indicators (such as titratable acidity, tartaric acid, citric acid, and acetic acid) contributed differently to PC1 and PC2, with varying positive and negative loading values, indicating that various acids had different impacts on the quality of grape juice. 

Overall, Kyoho was closer to the flavor properties typically found in commercial Concord grape juice compared to the other varieties. Kyoho was grouped together with Seto Giant, Black Swan, Gold Finger, and High Bailey based on aromatic profiles of 20 different Chinese table grapes [32]. The between-group-discriminating aroma descriptors included fatty, balsamic, herbaceous, floral, fruity, and sweet [32], among which floral and fruity compounds were also involved in the classification of the five grapes in this study.

#### 3.2.4. Summary of Aroma Properties

Concord and Kyoho exhibited the highest aroma intensity, followed by Summer Black and Moldovan, and all displayed floral and fruity notes mainly contributed by norisoprenoids or esters. Sweet Sapphire possessed the lowest aroma intensity and emitted a grassy scent. Concord featured foxy notes by methyl anthranilate, which was not found in any of the other four Chinese grapes.

In summary, Concord and Kyoho had similar flavor compositional profiles, while Sweet Sapphire, Summer Black, and Moldovan were classified together. This classification was differentiated by aroma and taste. Concord and Kyoho had a fruitier and more floral aroma as well as a more balanced sweet–sour ratio. Sweet Sapphire, Summer Black, and Moldovan had fewer aroma compounds and a sweeter taste. Therefore, Kyoho would be the most suitable variety for making grape juice based on flavor properties. 

### 3.3. Color Property Analysis

#### 3.3.1. Monomeric Anthocyanins and Anthocyanin Profile

Color, as a pivotal sensory attribute of food, influences consumer perception and preference. Anthocyanins are the primary pigment in grapes, and their content (mainly in the monomeric form) has a direct impact on the color of grape juice. Besides anthocyanin content, color is influenced by various factors including anthocyanin profile (distinguished by their anthocyanidin, acylation, glycosylation, and cis-trans isomerism), the pH value (impacting the acid–base and hydration equilibria of flavylium cation), and co-pigmentation effects (self-association, co-pigmentation, and metal binding) [52,53,54].

**Figure 3:**
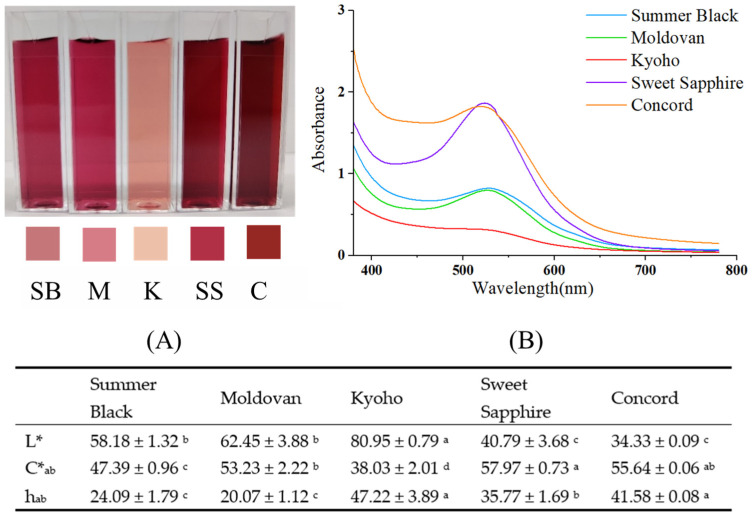
Color (**A**) and spectrum at a wavelength range of 380–780 nm (**B**) of five grape varieties. SB = Summer Black, M = Moldovan, K = Kyoho, SS = Sweet Sapphire, C = Concord. Significant differences at the *p* < 0.05 level are denoted by distinct lowercase letters within the same row.

Figure 3 illustrates the colors and spectra of the five grape juice varieties, revealing significant variations in color. Kyoho exhibited a notably lighter color than all other varieties with the lowest absorbance at λmax (Figure 3B). Sweet Sapphire, on the other hand, showed the most intense red color among all samples. Aside from C*_ab_, no significant differences in color parameters (L*, h_ab_) were observed between Summer Black and Moldovan. Concord, with a monomeric anthocyanin content of 61.11 mg cyanidin 3-glucoside equivalent/L, lower than that of Sweet Sapphire, Summer Black, and Moldovan (Table 5), unexpectedly exhibited the second most intense color (Figure 3), presenting an intriguing phenomenon that warrants further investigation.

There were significant differences in monomeric anthocyanin content among the five grape varieties, with a range from 27 to 218 mg cyanidin 3-glucoside equivalent/L (Table 5). Sweet Sapphire exhibited the highest monomeric anthocyanin content, whereas that of Kyoho was significantly lower than for all other varieties. In terms of anthocyanin profiles, the elution order and mass spectrometry patterns of anthocyanin in this study (Figure S1, Table S1) generally aligned with previous research, despite minor variations in identified anthocyanin species [55,56,57,58,59]. Concord, as a common table grape, has had its anthocyanin profiles studied previously. The three major anthocyanins in Concord grape were delphinidin 3-glucoside, cyanidin 3-glucoside, and delphinidin 3-coumaroyl-glucoside [22]. Besides these three anthocyanins, delphinidin (Dp), cyanidin (Cy), petunidin (Pt), peonidin (Pn), and malvidin (Mv) derivatives with glucoside, diglucoside, coumaroyl-glucoside, acetyl-glucoside, and coumaroyl-diglucoside were also found in Concord grape. In our study, the Concord grape anthocyanin profile matched with that of this study. Prior studies also reported trace amounts of pelargonidin (Pg) and pentose-anthocyanins in Concord [22], which were not detected in our study. Summer Black and Moldovan had similar anthocyanin profiles. Mv derivatives were the dominant anthocyanins in these two grapes followed by Pn derivatives. Similar results were also reported by Zhang et al. [59] in grape skin. Previous research also found a relatively large amount of Pt derivatives and trace amounts of Dp, Pg, and Cy derivatives in Summer Black grape skin [59]. Petunidin 3-glucoside was only found in Moldovan in our study. For Kyoho, the major anthocyanins in fresh skin were malvidin 3-(trans)-coumaroyl-5-diglucoside, malvidin 3-glucoside, and delphinidin 3-glucoside reported by Li et al. [60]. However, we did not detect a large amount of these three anthocyanins in Kyoho grape juice. Malvidin 3,5-diglucoside and peonidin 3,5-diglucoside were the major anthocyanins found in Kyoho grape juice. For the Sweet Sapphire grape, malvidin and peonidin 3-O-glucoside were the major anthocyanins which matched the results reported by Cruz et al. [61]. We identified caffeoyl-anthocyanins in four Chinese grape varieties, which have rarely been reported in those four grapes before. The detection of anthocyanin profile can be affected by the extraction method, different plant tissue, or the analytical method. In our project, we did not use any solvent such as acetone or methanol, which is more favorable to extract anthocyanin. In addition, since anthocyanins are distributed mostly in grape skin, anthocyanins might be diluted by the juice squeezed from flesh, which might result in trace amounts of anthocyanins undetected in our study.

Even though Concord grape juice showed significantly higher color intensity than Summer Black and Moldovan, the Concord monomeric anthocyanin concentration (61.11 mg/L) was significantly lower than that of Summer Black (134.34 mg/L) and Moldovan (124.32 mg/L). This could be explained by co-pigmentation between anthocyanin and phenolic compounds in grapes since Concord grapes had a significant higher phenolic concentration than three of the other grapes. Co-pigmentation, the molecular interaction between anthocyanins and co-pigments, is mediated by van der Waals forces, hydrophobic effects, and hydrogen bonding. This molecular interaction leads to the formation of a characteristic π–π stacking, which protects the flavonoid cation from hydration, consequently enhancing both the stability and intensity of the color. Grape juice contains various phenolic acids such as caffeic acid, ferulic acid, p-coumaric acid, and gallic acid, which are commonly recognized as potential co-pigments [62,63]. The polymeric color percentages of the five grape varieties were evaluated to reflect the extent of polymeric anthocyanin formation and their contribution to the overall color stability. Concord exhibited the highest polymeric color percent (52.36%), followed by Summer Black (42.69%), Moldovan (40.79%), Kyoho (30.71%), and Sweet Sapphire (18.12%) (Table 5), suggesting polymeric anthocyanins contributed to some extent to Concord’s color. Note that Concord was the only juice that was pasteurized, and the heat treatment the Concord juice had undergone could have caused more anthocyanins to polymerize. Polymers also tend to have a more brownish hue, that could contribute to a higher hue value and more absorbance in the 440 nm region. The formation of polymeric anthocyanins might also play a role in the darker appearance of grape juice. 

In addition to co-pigmentation, Concord grape juice had the lowest pH (3.59 ± 0.03) among the grape juices evaluated. The coloration of anthocyanins is greatly influenced by pH. At pH ≤ 3 the red flavylium cations predominate. As pH rises, a kinetic and thermodynamic competition occurs between the hydration reaction and the proton transfer reactions. Within the pH range of 4 to 6, this competition primarily leads to the formation of the colorless chalcone [64,65]. Since a higher proportion of Concord pigments were in the colored flavylium form, Concord may present more vibrant color than other grape varieties.

**Figure 4:**
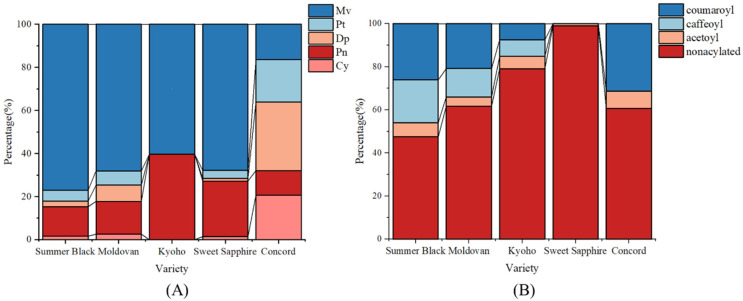
Anthocyanidin proportions (**A**) and acylated ratios (**B**) in five grape varieties. Mv = Malvidin, Pt = Petunidin, Dp = Delphinidin, Pn = Peonidin, Cy = Cyanidin.

Anthocyanidin type also affects color expression [66]. Figure 4A shows the anthocyanidin proportion in the five grapes. Malvidin was the predominant anthocyanidin in the four Chinese grapes while delphinidin was predominant in Concord grapes. In general, Dp tends to have more reddish color while Mv has a blueish hue. However, our results did not show a similar trend, which indicated other factors including pH, acylation, and glycosylation patterns should be taken into account.

Acylation can also significantly affect the color and stability of anthocyanins. Figure 4B shows the ratio of acylated pigments in the five grapes. Summer Black had the highest acylation ratio (52.5%), while Sweet Sapphire had the lowest (1%). The main types of acylation were identified in *p*-coumaric acid, caffeic acid, and acetic acid. These identifications are tentative, based on retention time, order of elution, spectra, mass, MS/MS, and comparison to the literature. The acylating groups can protect one side of the pyrylium ring from nucleophilic attack, thereby increasing the stability of the anthocyanin and its color [67].

#### 3.3.2. Summary of Color Properties

The color intensities of Sweet Sapphire and Concord were the highest, followed by Summer Black and Moldovan, whereas Kyoho displayed the lowest chroma. Mv emerged as the predominant anthocyanin in all four Chinese grape varieties while Dp was the primary anthocyanin in Concord. Sweet Sapphire showed the greatest color intensity but it contained negligible amounts of acylated anthocyanins, suggesting relatively lower color stability during processing and shorter shelf life. Therefore. Summer Black could be a good source to provide color for grape juice.

## 4. Conclusions

The four grape varieties demonstrated significant disparities in physicochemical, aroma component, and color properties. Juicing grapes ought to possess characteristics such as high juice yield, optimal Brix–acid ratio, rich aroma, and vivid color. Sweet Sapphire and Kyoho yielded the highest amount of juice in all grapes. Sweet Sapphire and Concord had the best sugar–acid ratios of all. The Brix–acid ratio of Summer Black and Moldovan exceeded 60, leading to an over-sweet taste, whereas Kyoho’s ratio was around 27, resulting in a slightly sour taste. Regarding aroma, Concord and Kyoho possessed the richest aroma, with the floral and fruity β-damascenone identified as one of the key aroma contributors. A total of 16 aroma-active compounds were identified, with the fruity, sweet, and floral ethyl acetate, 1-hexanol, and linalool found in all five grape varieties. Sweet Sapphire emitted a slightly grassy scent, whereas the other four grape varieties emanated rosy and fruity notes. Sweet Sapphire and Concord demonstrated the highest color intensity with a winey red hue, whereas Summer Black and Moldovan displayed a bright red color, and Kyoho appeared pinkish brown. Though Sweet Sapphire had the highest pigment content, its low acylation ratio suggests limited processing stability. Summer Black featured a vibrant color and it contained 52.5% acylated anthocyanins, making it a competitive choice as an appealing juice ingredient. Since Kyoho provided the most similar aroma profiles to the commercially available Concord grape juice and Summer Black had vibrant and stable color and anthocyanin profiles, a mixture of Kyoho and Summer Black grape juice could be formulated to provide all-round sensory quality. In addition to the aroma and color, Summer Black could provide a sweet taste and complement the sour notes of Kyoho grapes. Future research could focus on sensory analysis of juice prepared from a mixture of these two grapes in different ratios to better meet consumers’ expectations.

## Figures and Tables

**Table 1:** Four Chinese purple table grapes’ information.

Name	Summer Black	Moldovan	Kyoho	Sweet Sapphire
Picture	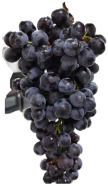	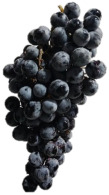	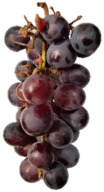	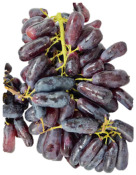
Classification	*Vitis vinifera* × *Vitis labrusca*	*Vitis vinifera* × *Vitis labrusca*	*Vitis vinifera* × *Vitis labrusca*	*Vitis vinifera*
Producing area	Bortala Mongol Autonomous Prefecture, Xinjiang Uygur Autonomous Region, China	Bortala Mongol Autonomous Prefecture, Xinjiang Uygur Autonomous Region, China	Shenyang City, Liaoning Province, China	Shijiazhuang City, Hebei Province, China
Harvest time	September 2023	September 2023	January 2024	January 2024

**Table 2:** Physicochemical properties of five grape varieties.

	Summer Black	Moldovan	Kyoho	Sweet Sapphire	Concord
Yield (%)	69.88 ± 1.39 ^b^	74.03 ± 2.25 ^ab^	75.38 ± 1.91 ^a^	76.06 ± 2.52 ^a^	-
pH	3.99 ± 0.02 ^a^	3.84 ± 0.04 ^b^	3.68 ± 0.02 ^cd^	3.78 ± 0.07 ^c^	3.59 ± 0.03 ^d^
Soluble solids (°Brix)	22.8 ± 0.4 ^a^	21.8 ± 0.5 ^a^	14.6 ± 0.4 ^d^	18.2 ± 0.5 ^b^	16.3 ± 0.1 ^c^
Titratable acidity (g/L)	3.20 ± 0.21 ^c^	3.26 ± 0.17 ^c^	5.27 ± 0.56 ^a^	4.17 ± 0.24 ^b^	5.15 ± 0.07 ^a^
Brix–acid ratio	71.66 ± 5.80 ^a^	66.87 ± 3.77 ^a^	27.84 ± 2.02 ^c^	43.65 ± 1.39 ^b^	31.56 ± 0.41 ^c^
Sucrose (g/L)	ND	ND	ND	ND	ND
Fructose (g/L)	89.71 ± 7.70 ^a^	89.46 ± 0.23 ^a^	68.87 ± 8.31 ^b^	91.14 ± 0.86 ^a^	60.88 ± 1.22 ^b^
Glucose (g/L)	64.89 ± 4.00 ^a^	64.80 ± 0.68 ^a^	36.90 ± 3.90 ^b^	56.84 ± 0.75 ^a^	36.58 ± 0.80 ^b^
Sweetness index	204.81 ± 11.74 ^a^	205.14 ± 0.08 ^a^	148.19 ± 12.35 ^b^	202.13 ± 0.67 ^a^	133.98 ± 2.66 ^b^
Ascorbic acid (mg/L)	0.033 ± 0.009 ^cd^	0.021 ± 0.008 ^d^	0.059 ± 0.011 ^b^	0.093 ± 0.011 ^a^	0.056 ± 0.004 ^bc^
Oxalic acid (g/L)	0.50 ± 0.01 ^a^	0.36 ± 0.00 ^c^	0.25 ± 0.01 ^d^	0.43 ± 0.02 ^b^	0.33 ± 0.00 ^c^
Tartaric acid (g/L)	19.26 ± 0.11 ^a^	16.91 ± 0.69 ^b^	14.02 ± 0.97 ^c^	14.38 ± 0.64 ^c^	4.67 ± 0.01 ^d^
Malic acid (g/L)	1.58 ± 0.05 ^cd^	1.05 ± 0.09 ^d^	2.41 ± 0.28 ^ab^	2.04 ± 0.37 ^bc^	2.80 ± 0.01 ^a^
Acetic acid (g/L)	0.85 ± 0.06 ^b^	0.74 ± 0.07 ^b^	0.73 ± 0.05 ^b^	0.54 ± 0.03 ^c^	1.25 ± 0.01 ^a^
Citric acid (g/L)	0.50 ± 0.07 ^b^	0.13 ± 0.02 ^d^	0.95 ± 0.08 ^a^	0.20 ± 0.04 ^cd^	0.31 ± 0.00 ^c^
Succinic acid (g/L)	0.59 ± 0.01 ^a^	0.27 ± 0.02 ^b^	0.24 ± 0.06 ^b^	0.30 ± 0.05 ^b^	0.07 ± 0.00 ^c^

Note: The data presented represent mean values (n = 3). Significant differences at the *p* < 0.05 level are denoted by distinct lowercase letters within the same row. Titratable acidity was calculated using tartaric acid as the equivalent. “ND” meant the compound was not detected by the instrument.

**Table 3:** Class of volatiles and their concentrations in five grape varieties.

	Summer Black	Moldovan	Kyoho	Sweet Sapphire	Concord
Total volatiles (μg/L)	879.97 ± 92.80 ^b^	641.45 ± 106.90 ^bc^	1972.55 ± 205.72 ^a^	409.02 ± 87.40 ^c^	1774.61 ± 221.10 ^a^
Alcohols (μg/L)	317.50 ± 40.54 ^a^	200.43 ± 30.12 ^b^	134.72 ± 35.82 ^b^	206.46 ± 54.17 ^b^	184.58 ± 8.71 ^b^
Terpenes (μg/L)	145.07 ± 18.69 ^a^	97.04 ± 18.12 ^b^	3.21 ± 0.34 ^c^	7.51 ± 2.72 ^c^	15.93 ± 0.40 ^c^
Aldehydes (μg/L)	151.00 ± 15.27 ^ab^	120.87 ± 30.48 ^ab^	100.13 ± 9.39 ^b^	168.8 ± 33.11 ^a^	23.55 ± 1.38 ^c^
Ketones (μg/L)	2.91 ± 1.06 ^b^	2.10 ± 0.23 ^b^	3.62 ± 0.83 ^b^	4.62 ± 2.23 ^b^	63.49 ± 9.73 ^a^
Acids (μg/L)	3.08 ± 0.20 ^a^	3.10 ± 0.64 ^a^	5.11 ± 1.35 ^a^	3.25 ± 1.02 ^a^	3.36 ± 0.40 ^a^
Esters (μg/L)	173.94 ± 53.73 ^b^	167.80 ± 25.34 ^b^	1679.05 ± 176.31 ^a^	5.88 ± 3.61 ^b^	1461.95 ± 203.08 ^a^
Aromatics (μg/L)	84.23 ± 7.99 ^a^	48.38 ± 10.07 ^b^	42.88 ± 9.52 ^b^	12.5 ± 1.93 ^c^	18.72 ± 0.21 ^c^
Norisoprenoids (μg/L)	ND	ND	3.82 ± 0.65 ^a^	ND	3.04 ± 0.16 ^b^
Others (μg/L)	2.24 ± 0.17 ^a^	1.74 ± 0.22 ^b^	ND	ND	ND

Note: The data presented represent mean values (n = 3). Significant differences at the *p* < 0.05 level are denoted by distinct lowercase letters within the same row. “ND” indicates that the compound was not detected in the analysis.

**Table 4:** Odor activity values (OAVs) of aroma compounds identified in five grape varieties.

Compounds	Threshold	Summer	Moldovan	Kyoho	Sweet	Concord
	(μg/kg)	Black	OAVs	OAVs	Sapphire	OAVs
		OAVs			OAVs	
**Esters**						
Ethyl acetate	5	32.1	31.8	311	1.18	278
Ethyl butyrate	0.9	ND	ND	115	ND	48.3
Ethyl hexanoate	2.2	ND	ND	4.70	ND	0.685
Ethyl 2-hexenoate	no reference	ND	ND	no reference	ND	ND
Methyl (*S*)-(+)-3-hydroxybutyrate	no reference	ND	ND	ND	ND	no reference
Ethyl 3-hydroxybutyrate	2500	0.00282	0.00143	0.00196	ND	0.00186
Ethyl benzoate	55.56	ND	ND	0.0205	ND	ND
Ethyl 3-hydroxyhexanoate	45	ND	ND	0.0115	ND	ND
Methyl salicylate	40	0.154	0.129	ND	ND	ND
Ethyl 2-phenylacetate	155.55	ND	ND	0.0149	ND	0.0108
Phenethyl acetate	250	ND	ND	ND	ND	0.00740
Methyl anthranilate	3	ND	ND	ND	ND	4.62
Subtotal		32.3	31.9	431	1.18	332
%		22.97%	26.43%	18.25%	1.79%	17.50%
**Aldehydes**						
Isovaleraldehyde	6.1	ND	1.43	ND	ND	ND
Hexanal	5	19.8	15.1	6.84	19.2	ND
(*E*)-2-hexenal	88.7	0.567	0.474	0.743	0.316	0.0239
1-Nonanal	1.1	ND	1.05	ND	ND	ND
Furfural	9562	ND	ND	ND	ND	0.00224
(*Z*)-3,7-Dimethylocta-2,6-dienal	53	0.0197	0.0146	ND	ND	ND
Subtotal		20.4	18.1	7.58	19.5	0.0261
%		14.52%	14.96%	0.32%	29.63%	0.00%
**Aromatics**						
Styrene	3.6	ND	ND	0.191	0.812	ND
*α*, *p*-Dimethylstyrene	85	0.0188	0.0136	0.00777	ND	ND
Benzaldehyde	750.89	0.00342	0.00256	0.00303	0.00241	0.0156
Phenylacetaldehyde	2.2	1.08	1.15	3.42	2.13	ND
Acetophenone	65	ND	ND	ND	ND	0.0185
2,4-Dimethylbenzaldehyde	no reference	no reference	no reference	ND	ND	ND
Benzyl alcohol	2546.21	0.00368	0.00266	0.000294	0.000752	ND
Phenethyl alcohol	564.23	0.116	0.0588	0.0549	0.00205	0.0102
4-Isopropylbenzyl alcohol	no reference	no reference	no reference	ND	ND	ND
Subtotal		1.22	1.23	3.68	2.95	0.0443
%		0.87%	1.02%	0.16%	4.47%	0.00%
**Ketones**						
Acetone	832	ND	ND	ND	ND	0.0745
Acetoin	14	ND	ND	0.259	0.330	ND
(*R*)-(-)-6-Methyl-5-hepten-2-ol	68	0.00148	0.000850	ND	ND	ND
6-Methyl-5-hepten-2-one	2000	0.0336	0.0308	ND	ND	ND
4-Methoxy-2,5-dimethyl-3 (*2H*)-furanone	160	ND	ND	ND	ND	0.00965
Subtotal		0.0351	0.0316	0.259	0.330	0.0842
%		0.02%	0.03%	0.01%	0.50%	0.00%
**Alcohols**						
Ethanol	95000	0.000197	0.0000783	0.000109	0.0000457	ND
2-Methyl-3-buten-2-ol	1140	ND	0.0246	ND	ND	ND
3-Methyl-2-butanol	820	ND	ND	ND	0.0164	ND
3-Methyl-1-butanol	6.1	ND	2.78	ND	8.89	2.12
3-Methyl-3-buten-1-ol	547.125	ND	0.0184	ND	0.00575	0.00598
3-Methyl-2-buten-1-ol	250	0.0648	0.0381	0.0104	ND	0.0212
1-Hexanol	5.6	14.7	6.92	2.73	19.8	14.9
(*E*)-3-Hexen-1-ol	110	ND	ND	ND	0.0494	0.0146
(*Z*)-3-Hexen-1-ol	3.9	2.55	1.97	0.432	0.761	1.50
(*E*)-2-Hexen-1-ol	3593	0.0404	0.0140	0.00145	0.00367	0.0162
1-Octen-3-ol	1.5	ND	1.39	1.34	0.745	1.18
1-Heptanol	5.4	0.300	ND	ND	ND	0.461
2-Ethylhexanol	25482.2	0.000225	0.000131	0.000115	0.000115	0.00535
1-Octanol	125.8	0.0148	0.00901	0.00869	0.00542	0.0440
1-Decanol	2800	ND	ND	ND	ND	0.000780
Subtotal		17.7	13.2	4.52	30.3	20.3
%		12.58%	10.90%	0.19%	45.97%	1.07%
**Terpenes**						
*R*-(+)-Dipentene	34	0.582	0.343	ND	ND	0.0129
β-Ocimene	10	0.212	0.133	ND	ND	ND
Linalool	0.22	49.0	40.2	3.49	8.16	22.4
1-Terpinen-4-ol	1200	0.00253	0.00158	0.000759	0.000502	0.00139
α-Terpineol	1200	0.00488	0.00399	0.000646	0.000694	0.00127
Citral	120	0.0200	0.00911	ND	ND	ND
Citronellol	2200	0.0127	0.0128	0.000342	ND	ND
Nerol	53	0.0248	0.0107	ND	0.000219	ND
Geraniol	1.1	16.9	14.3	ND	3.45	ND
Subtotal		66.8	55.0	3.49	11.6	22.4
%		47.54%	45.54%	0.15%	17.63%	1.18%
**Norisoprenoid**						
β-Damascenone	0.002	ND	ND	1910	ND	1520
Subtotal		ND	ND	1910	ND	1520
%		0%	0%	80.92%	0%	80.23%
**Acid**						
Acetic acid	99000	0.0000311	0.0000313	0.0000516	0.0000328	0.0000339
Subtotal		0.0000311	0.0000313	0.0000516	0.0000328	0.0000339
%		0.00%	0.00%	0.00%	0.00%	0.00%
**Others**						
(2*S*,4*R*)-*cis*-4-methyl-2-(2-methyl-1-propenyl) tetrahydropyran	0.5	2.11	1.37	ND	ND	ND
Dimethylmethylenepyran	no reference	no reference	no reference	ND	ND	ND
Subtotal		2.11	1.37	ND	ND	ND
%		1.50%	1.13%	0%	0%	0%
Total		140.44	120.81	2360.28	65.86	1894.46

Note: The odor thresholds of aroma compounds were derived from the “Odour Thresholds Compilations of Odour Thresholds” [18] dataset. The data presented represent mean values (n = 3). “ND” indicates that the compound was not detected in the analysis. “No reference” signifies that the odor threshold for the compound was not found in the available literature. The percentage calculation for each class of aromatic compounds was performed by dividing the OAVs of each compound by the total OAVs of all aromatic compounds. Gray shading is used to highlight values greater than or equal to 1.

**Table 5:** Monomeric anthocyanin contents and major anthocyanins profile (concentrations were calculated based on percent area for leaks >5% at 520 nm) in five grape varieties.

Pigments and Co-Pigments *	Summer Black	Moldovan	Kyoho	Sweet Sapphire	Concord
Concentration (mg cyanidin 3-glucoside equivalent/L)					
Total monomeric anthocyanin	134.34 ± 7.36 ^b^	124.32 ± 2.78 ^b^	27.08 ± 3.16 ^d^	218.56 ± 15.04 ^a^	61.11 ± 3.40 ^c^
Delphinidin 3-glucoside	-	-	-	-	6.86
Delphinidin 3-coumaroyl-5-diglucoside	-	-	-	-	5.19
Cyanidin 3-glucoside	-	-	-	-	5.32
Cyanidin 3-coumaroyl-5-diglucoside	-	-	-	-	3.38
Petunidin 3-glucoside	-	6.69	-	-	-
Petunidin 3,5-diglucoside	-	-	-	-	4.84
Petunidin 3-coumaroyl-5-diglucoside	-	-	-	-	3.08
Peonidin 3,5-diglucoside	8.41	9.95	6.06		4.52
Peonidin 3-glucoside	-	-	2.86	55.73	-
Peonidin 3-coumaroyl-5-diglucoside	6.85	-	-	-	-
Malvidin 3,5-diglucoside	16.30	14.16	6.28	-	-
Malvidin 3-glucoside	22.99	25.66	5.61	144.01	-
Malvidin 3-caffeoyl-5-diglucoside	22.53	13.02	1.52	-	-
Malvidin 3-coumaroyl-5-diglucoside	23.56	17.67	-	-	4.85
Co-pigment concentration (mg gallic acid equivalent/L)					
Total phenolics	1053.57 ± 3.26 ^c^	894.56 ± 8.69 ^d^	535.85 ± 42.87 ^e^	1840.75 ± 71.53 ^a^	1431.49 ± 64.09 ^b^
Polymeric color (%)	42.69 ± 2.00 ^b^	40.79 ± 0.51 ^b^	30.71 ± 7.11 ^c^	18.12 ± 1.15 ^d^	52.36 ± 1.69 ^a^

Note: Significant differences at the *p* < 0.05 level are denoted by distinct lowercase letters within the same row. Monomeric anthocyanin was calculated using cyanidin 3-glucoside as the equivalent. Total phenolics was calculated using gallic acid as the equivalent. The monomeric anthocyanin content is multiplied by the peak area percentage at 520 nm to obtain the content of each anthocyanin. * Tentative identification based on retention time, order of elution, spectra, mass, MS/MS, and comparison to the literature.

## Data Availability

The original contributions presented in the study are included in the article/Supplementary Materials, further inquiries can be directed to the corresponding authors.

## Supplementary Materials

The following supporting information can be downloaded at: https://www.mdpi.com/article/10.3390/foods13233889/s1, Figure S1: Chromatograms of anthocyanin from five grape varieties. Overlapped peaks in the chromatogram were converted based on the mass spectrometry area; Table S1: Major anthocyanin compounds (percentage > 5% in 520 nm) of five grape varieties; Table S2: Standard curve.
