# Supplementary material for: A Comparative Study of Physicochemical, Aroma, and Color Profiles Affecting the Sensory Properties of Grape Juice from Four Chinese Vitis vinifera × Vitis labrusca and Vitis vinifera Grapes
Source: Foods. 2024 Dec 2;13(23):3889. doi: 10.3390/foods13233889 (PMC11640426; doi:10.3390/foods13233889)
Supplement: Supplementary file 1 [file foods-13-03889-s001.zip › foods-3303837-supplementary.pdf]

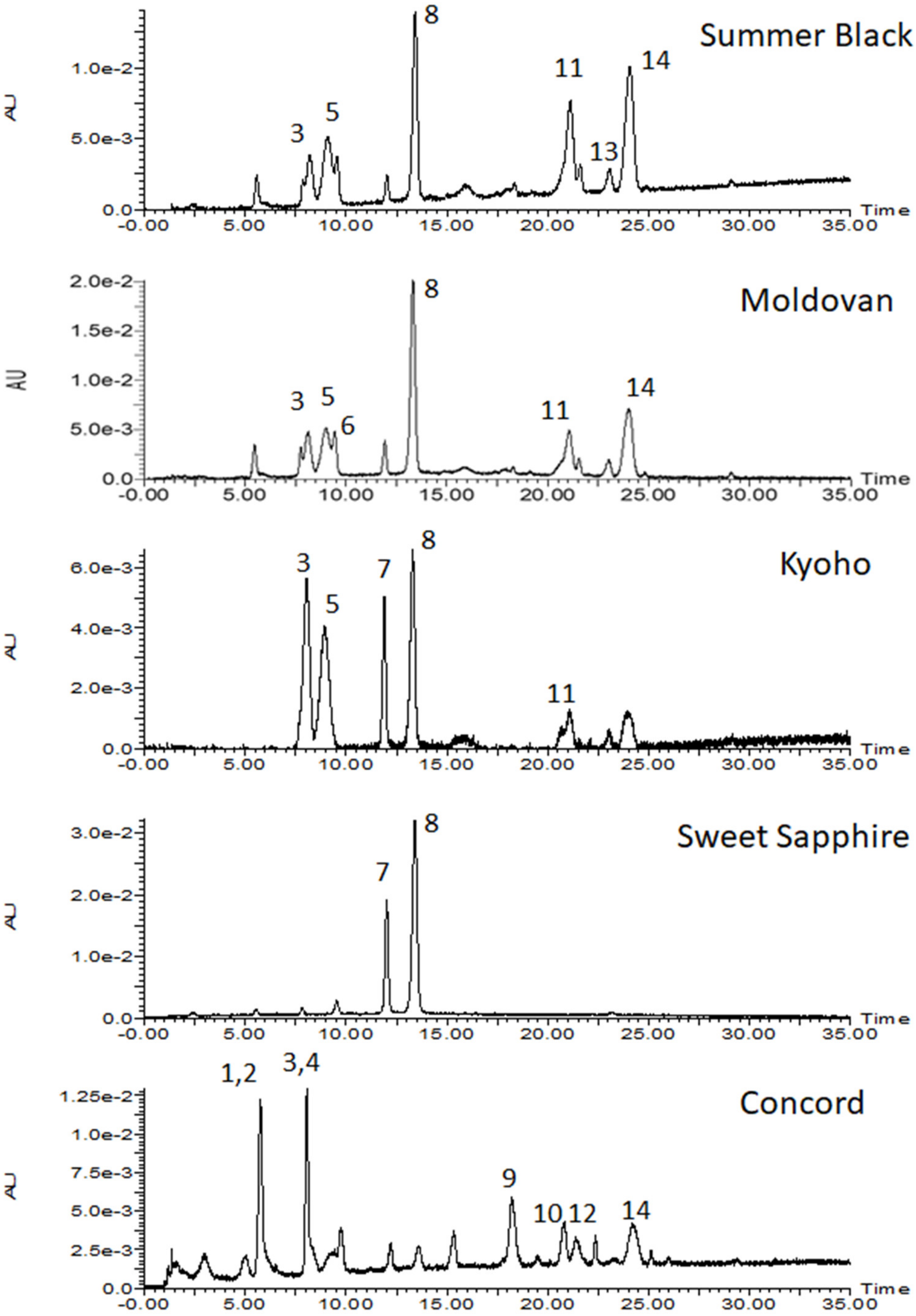

Figure S1. Chromatograms of anthocyanin from five grape varieties. Overlapped peaks in the chromatogram were converted based on the mass spectrometry area.

Table S1. Major anthocyanin compounds (percentage > 5% in 520 nm) of five grape varieties.

No.	Retention time (min)	λmax (nm)	[M] (m/z+)	MS^2 (m/z)	Natural Loss	Compounds
1	5.80	520	465	303	162	delphinidin 3-glucoside
2	6.12	521	641	317	324	petunidin 3,5-diglucoside
3	8.06	514	625	301	324	peonidin 3,5-diglucoside
4	8.20	516	449	287	162	cyanidin 3-glucoside
5	9.00	523	655	331	324	malvidin 3,5-diglucoside
6	9.59	524	479	317	162	petunidin 3-glucoside
7	12.14	512	463	301	162	peonidin 3-glucoside
8	13.53	525	493	331	162	malvidin 3-glucoside
9	18.19	528	773	303	470	delphinidin 3-(coumaroyl)-5-diglucoside
10	20.89	527	757	287	470	cyanidin 3-(coumaroyl)-5-diglucoside
11	21.23	529	817	331	486	malvidin 3-(caffeoyl)-5-diglucoside
12	21.38	530	787	317	470	petunidin 3-(coumaroyl)-5-diglucoside
13	23.92	529	771	301	470	peonidin 3-(coumaroyl)-5-diglucoside
14	24.41	530	801	331	470	malvidin 3-(coumaroyl)-5-diglucoside

Table S2. Standard curve.

Compounds	Standard curve	R^2
Gallic acid	y = 0.1019 x + 0.0413	0.9990
Sucrose	y = 5106090 x - 49349	0.9933
Glucose	y = 3440186 x - 97918	0.9960
Fructose	y = 5095237 x + 643961	0.9915
Ascorbic acid	y = 65444x + 6253.1	0.9999
Oxalic acid	y = 16452578 x - 10984	1.0000
Tartaric acid	y = 458984 x - 24802	0.9975
Malic acid	y = 1000497 x - 53573	0.9973
Acetic acid	y = 624934x - 1934.8	1.0000
Citric acid	y = 1041228 x - 462	1.0000
Succinic acid	y = 213491 x - 3598	0.9983
